# Early Radiation-Induced Sarcoma in an Adolescent Treated for Relapsed Hodgkin Lymphoma with Nivolumab

**DOI:** 10.3390/medicina56040155

**Published:** 2020-03-31

**Authors:** Lukas Šalaševičius, Goda Elizabeta Vaitkevičienė, Ramunė Pasaulienė, Rosita Kiudelienė, Ernesta Ivanauskaitė-Didžiokienė, Donatas Vajauskas, Nemira Jurkienė, Jelena Rascon

**Affiliations:** 1 Faculty of Medicine, Vilnius University, 03101 Vilnius, Lithuania; lukassalasevicius@gmail.com (L.Š.); GodaElizabeta.Vaitkeviciene@santa.lt (G.E.V.); 2 Center for Pediatric Oncology and Hematology, Vilnius University, 08406 Vilnius, Lithuania; ramune.pasauliene@santa.lt; 3 Center of Pediatric Oncology and Hematology at Pediatric Department and Hospital of Kauno Klinikos, Lithuanian University of Health Sciences, 44307 Kaunas, Lithuania; rosita.kiudeliene@kaunoklinikos.It; 4 National Center of Pathology, 08406 Vilnius, Lithuania; Ernesta.IvanauskaiteDidziokiene@vpc.lt; 5 Institute of Biomedical Sciences, Department of Radiology, Nuclear Medicine and Medical Physics, Vilnius University, 03101 Vilnius, Lithuanian; donatas.vajauskas@kaunoklinkikos.lt; 6 Radiology and Nuclear Medicine Center, Department of Nuclear Medicine, Vilnius University Hospital Santaros Klinikos, 08661 Vilnius, Lithuania; 7 Radiology Clinic, Nuclear Medicine Department of Kauno Klinikos, Lithuanian University of Health Sciences, 44307 Kaunas, Lithuania; nemira.jurkiene@kaunoklinikos.lt

**Keywords:** Hodgkin lymphoma, post-radiation sarcoma, Nivolumab, children, obesity

## Abstract

Radiation-induced sarcoma (RIS) has been reported as a late secondary malignancy following radiotherapy for various types of cancer with a median latency of 10 years. We describe an early RIS that developed in an adolescent within three years of treatment (including PD-L1 check-point inhibitor Nivolumab) of a relapsed classic Hodgkin lymphoma (HL) and was diagnosed post-mortem. The patient died of the progressive RIS that was misleadingly assumed to be a resistant HL based on the positive PET/CT scan. Repetitive tumor biopsies are warranted in cases of aggressive and multi-drug resistant HL to validate imaging findings, ensure correct diagnosis and avoid overtreatment.

## 1. Introduction

Current five-year overall survival in pediatric Hodgkin lymphoma (HL) exceeds 90% [1]. However, secondary malignant neoplasms (SMNs) are the most relevant long-term sequelae comprising of 75%–80% of late effects [2]. Median latency period of SMNs development varies from 10 to 17.5 years [2,3]. Radiotherapy is one of the main risk factors for development of SMNs, often arising in the radiation field [3]. Even low-dose irradiation harbors a significant risk for SMNs with a cumulative incidence of 17% at 20 years [4]. Cancer of the breast, lung, and thyroid of various morphologies are the most common radiotherapy associated SMNs [5,6,7]. Secondary sarcomas are considered to be rare. Several small series reported radiotherapy associated sarcoma diagnosed 4 to 31 years following pediatric HL [4,8,9]

We describe a case of a relapsed classic HL that was treated with several lines of chemotherapy including PD-L1 check-point inhibitor Nivolumab. An early radiation-induced sarcoma (RIS) developed within three years after initial presentation and was diagnosed only post-mortem. Written informed consent from both parents was obtained before writing this study.

## 2. Case Report

**Figure 1:**
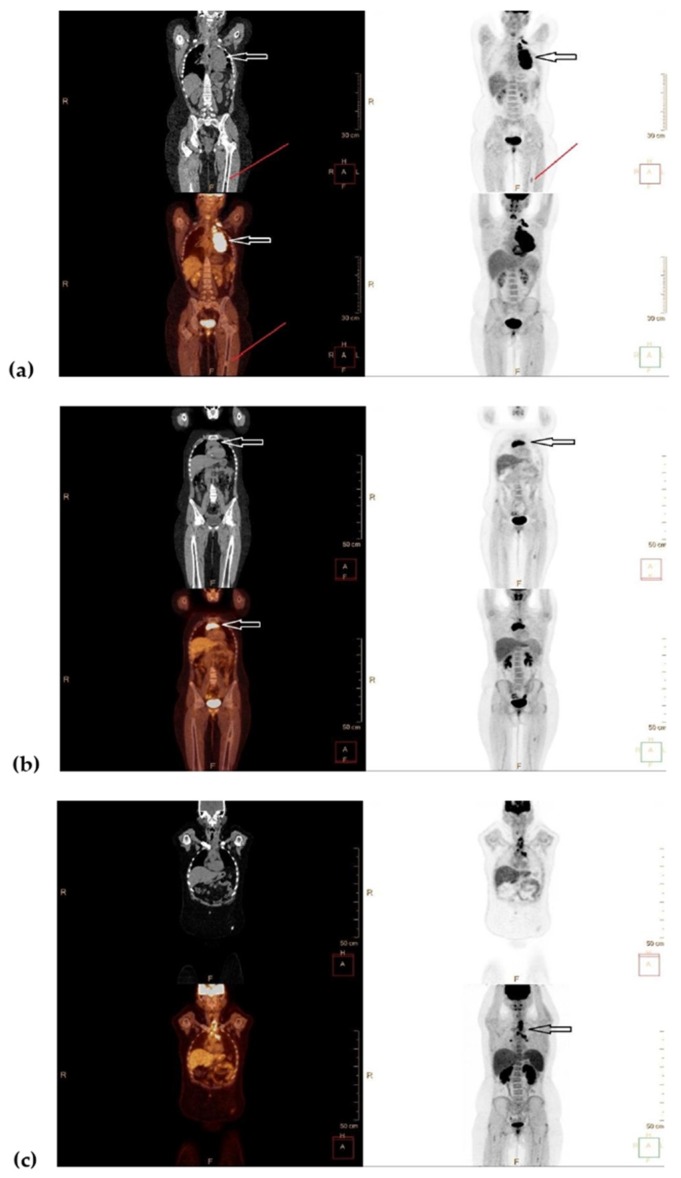
Whole body ^18^FDG PET/CT. (**a**) At diagnosis. Left supraclavicular and mediastinal ^18^FDG-avid lesions (arrows), ^18^FDG-avid lesion in the left femur (red line), according to lymphoma staging Lugano IV; (**b**) Relapse. ^18^FDG-avid relapse in the mediastinum (arrows). According to lymphoma response, Deauville 5b. No metabolic changes in the left femur after the biopsy of enchondroma; (**c**) One month prior to death. ^18^FDG-avid lesions in neck, mediastinum, and hilar lymph nodes bilaterally. According to lymphoma response, Deauville 4.

**Figure 2:**
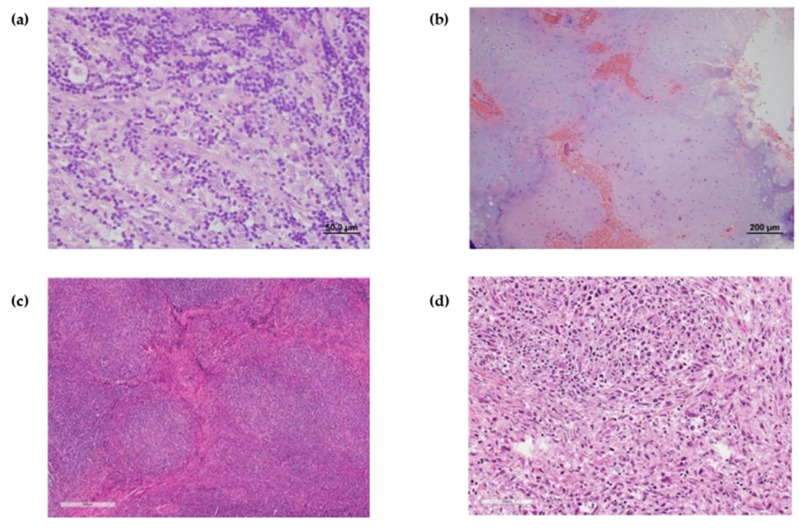
Pathology. (**a**) Diagnostic lymph node biopsy: classic Hodgkin lymphoma, nodular sclerosis, immune phenotype CD30+, CD15+, CD20/CD−. (**b**) Enchondroma on the left femur. (**c**) Relapse lymph node specimen: classic Hodgkin lymphoma, nodular sclerosis, immune phenotype CD30+, CD15+, CD20/CD3−, EBV LMP1−, ALK1−. (**d**) Autopsy. Secondary undifferentiated sarcoma. The tumor is formed of nodules composed of epithelioid or spindle cells with eosinophilic cytoplasm and large, hyperchromic, round or irregular nuclei expressing CD30+, CD15+/−, CD20/CD3−, VIM+ immune phenotype.

A 13 year old girl started to complain of persistent cough. Her physical examination at diagnosis was unremarkable except for obesity: weight and height were over 95 age and gender-specific percentile and body mass index (BMI) was 31.0. Endocrinological work-up did not reveal any potential cause of obesity. Neither palpable lymph nodes nor B symptoms were present. Imaging studies including positron emission tomography–computed tomography with 18 fluorine labeled fluorodeoxyglucose (^18^FDG-PET/CT) revealed an ^18^FDG-avid, bulky mediastinal mass of 13 cm, enlarged periclavicular lymph nodes, and a focus in the left femur that showed a moderate ^18^FDG uptake (Figure 1a). The biopsy taken from a periclavicular lymph node confirmed a classic HL, nodular sclerosis subtype, expressing immune phenotype CD30+, CD15+, CD20/CD3 (Figure 2a).

**Figure 3:**
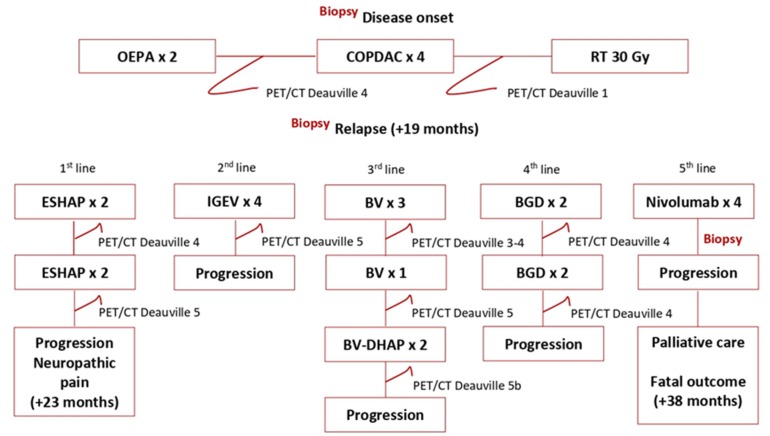
Disease evolution and treatment: BV—brentuximab vedotin 1.8 mg/kg every three weeks; COPDAC—prednisone 40 mg/m^2^ on days 1–15, dacarbazine 250 mg/m^2^ on days 1–3, vincristine 1.5 mg/m^2^ on days 1 and 15, cyclophosphamide 500 mg/m^2^ on days 1 and 8; DHAP—dexamethasone 40 mg on days 1–4, high-dose cytarabine 2.000 mg/m^2^ on day 2, cisplatin 100 mg/m^2^ on day 1; ESHAP—etoposide 40 mg/m^2^ on days 1–4, methylprednisolone 500 mg on days 1–5, cytarabine 2000 mg/m^2^ on day 5, cisplatin 25 mg/m^2^ on days 1–4; BGD—bendamustine 90 mg/m^2^ on days 1 and 2, gemcytabine gemcytabine 800 mg/m^2^ on days 1 and 4, dexamethasone 40 mg on days 1–4; IGEV—ifosfamide 2000 mg/m^2^ on days 1 and 4, vinorelbine 20 mg/m^2^ on day 1, gemcytabine 800 mg/m^2^ on days 1 and 4, prednisone 100 mg on days 1 and 4; OEPA—prednisone 60 mg/m^2^ on days 1–15, vincristine 1.5 mg/m^2^ on days 1, 8 and 15, doxorubicin 40 mg/m^2^ on days 1 and 15, etoposide 125 mg/m² on days 1–5; Nivolumab 240 mg every two weeks, RT—radiotherapy.

Taking the PET-positive focus in the left femur into consideration, the patient was assigned to the Lugano stage IV. Chemotherapy was initiated according to the EuroNet-PHL-C1 protocol as per stage IVa. Two OEPA (prednisone, vincristine, doxorubicin, etoposide) followed by 4 COPDAC (prednisone, dacarbazine, vincristine, cyclophosphamide) courses were successfully completed. An early response assessment by ^18^FDG-PET/CT after 2 OEPA courses showed Deauville 4 (Figure 3). After six chemotherapy cycles, a complete metabolic response was achieved (Deauville 2) with residual mediastinal mass of 6.2 × 8.6 cm. However, the focus in the left femur still showed metabolic activity with unchanged ^18^FDG uptake as compared to the diagnostic images. Radiotherapy of 30 Gy to the mediastinum was delivered. Thereafter, an overall unconfirmed complete remission (as per EuroNet-PHL-C1 protocol definition) was documented. A control ^18^FDG-PET/CT scan showed persistent moderate metabolic activity in the left femur. The biopsy taken from the femoral focus revealed a non-malignant enchondroma (Figure 2b).

Nineteen months after initial diagnosis a control magnetic resonance imaging (MRI) visualized an enlargement of the mediastinal masses. The biopsy showed the same histologic subtype of the classic HL-expressing diagnostic immune phenotype (Figure 2c). The PET/CT confirmed a ^18^FDG-avid relapse (Deauville 5b, Figure 1b). The patient received two ESHAP (etoposide, methylprednisolone, cytarabine, cisplatin) courses that decreased metabolic activity to Deauville 4 (Figure 3). After the third ESHAP cycle (23 months after the disease onset) the patient developed dyspnea and neuropathic pain in both arms due to compression of the brachial plexus. After the fourth ESHAP cycle, ^18^FDG-PET/CT showed new metabolically active masses in the neck and below the diaphragm and progressive disease in the mediastinum and bilaterally in the hilar lymph nodes (Deauville 5b).

Facing rapid disease progression, the second-line salvage therapy regimen IGEV (ifosfamide, gemcytabine, prednisone, vinorelbine) was administered. Following 4 cycles, the pain relieved, however, the control PET/CT showed no metabolic response, Deauville 4–5. The third-line salvage therapy of six cycles of Brentuximab vedotin (BV) was initiated. After four cycles of BV the ^18^FDG-PET/CT remained Deauville 5. Moreover, new foci with increased uptake in the mediastinum, lungs, and neck lymph nodes were documented. Given no metabolic response, DHAP (dexamethasone, high-dose cytarabine, cisplatin) chemotherapy was added to BV. After two cycles of immunochemotherapy, ^18^FDG-PET/CT remained Deauville 5. Therefore, the fourth salvage chemotherapy BGD (bendamustine, gemcytabine, dexamethasone) was administered (Figure 3). After two cycles, ^18^FDG-PET/CT revealed Deauville 4 (Figure 1c), however, after the third cycle, the patient experienced clinical and metabolic progression with newly onset fever, dysphagia, and pain in both arms.

Subsequently, Nivolumab was administered every two weeks (in total, four doses were infused). Before the second infusion, the patient developed an acute episode of air-way obstruction and asystolia provoked by dysphagia and choking. After resuscitation, persistent airway obstruction required intubation and prolonged ventilation, thus, an attempt of a tracheostomy was undertaken. The intervention was unsuccessful due to bulky tumor masses compressing the upper airways. The masses were partially excised: histologic evaluation showed no lymphoma cells in the tumor. Two days after the intervention, the patient passed away due to progressive airway obstruction (38th month of treatment). The autopsy confirmed the absence of HL cells and revealed an undifferentiated sarcoma infiltrating the neck, trachea, esophagus, and the naso- and oropharynx (Figure 2d). Extensive immune staining ruled out the classic HL and could not identify any specific type of sarcoma.

## 3. Discussion

Radio- and chemotherapy induced SMNs have a cumulative incidence rate of 19% after 30 years of diagnosis with a median latency period of 10 years [2,3]. Radiotherapy to the mediastinum of a cumulative dosage over 30 Gy is known to cause secondary cancer [5,6,10]. Our patient developed a secondary sarcoma (most probably radiation-induced) within 3 years after the initial diagnosis that is unusually early. The first two biopsies confirmed classic HL, therefore, RIS developed during the relapse therapy. It is impossible to determine the exact time-point of the tumor conversion as the imaging findings were unspecific for RIS or HL. ^18^FDG-PET/CT has demonstrated high negative-predictive value but substantially low positive-predictive value for prediction of outcomes in pediatric HL [11]. One can only speculate that neuropathic pain could be the first manifestation of RIS, which has a poor prognosis with estimated five-year survival of 12%–58% [12].

The patient was treated according to the EuroNet-PHL-C1 protocol where ^18^FDG-PET/CT is used to deliver a response-adapted therapy in an attempt to minimize exposure to radiotherapy. As per protocol, 30 Gy were delivered to the mediastinum based on the insufficient early response after two OEPA courses. A recent study confirmed an adverse prognostic value of a strongly enhanced residual ^18^FDG uptake in early PET-response for treatment outcome [13]. Adversely, the disease was overstaged to Lugano stage IV instead of II based on the positive ^18^FDG uptake in the benign enchondroma of the femur that caused initial overtreatment. A retrospective review of knee MRIs reported the prevalence of incidental enchodromas of 2.8%–2.9% in the adult population [14,15]. While benign bone tumors are less ^18^FDG-avid than malignant ones [16], enchondromas can demonstrate a high ^18^FDG uptake [17]. In our case, the enchondroma focus showed a stable moderate ^18^FDG uptake (as compared to fluctuant ^18^FDG-avid HL-derived lesions) that ultimately drove the decision to perform a biopsy.

Several studies in adults demonstrated an increased risk for HL development in the overweight population [18,19]. However, there are no convincing data whether high BMI compromises the disease outcome. Results of meta-analysis in children with hematological malignancies and osteosarcoma suggested that obesity was associated with worse survival: weight excess negatively affected the toxicity profile and increased the risk of treatment related mortality [20,21,22]. The impact of high BMI on disease progression in childhood leukemia remains unclear [20]. Some studies showed that excessive amounts of adipose tissue promoted resistances of leukemic cells to chemotherapy and facilitated tumor spread [21,23]. In contrast, in sarcoma patients, obesity did not appear to exacerbate the protumorigenic environment and aggravate a predisposition to tumor progression [24]. A recent case-control study suggested that obesity during childhood cancer treatment might be associated with increased risk for SMNs [25]. In view of scarce and inconsistent data, we can only speculate if obesity contributed negatively to the unfavorable outcome in our case.

A recent study on HL reported 5%–10% of patients will be refractory to initial treatment and 10%–30% will relapse after achieving an initial complete remission [26]. Despite multimodal treatment, the cure rate for relapsed or refractory disease does not exceed 49% [27]. In the adult population Nivolumab showed a remission rate of 65% in relapsed or progressive classic HL [28]. The evidence on efficacy in children is scarce. In our case, Nivolumab was administered as the fifth line of salvage therapy. It is impossible to prove whether Nivolumab or other cytotoxic drugs eradicated HL, which was undetectable on the third biopsy. The treatment was guided based on the response assessment on ^18^FDG-PET/CT. However, the ^18^FDG is a nonspecific radiotracer representing metabolic tissue activity that in our case was unspecific for HL, enchondroma, and RIS.

## 4. Conclusions

This case demonstrates that a SMN, in particular RIS, can develop early within the treatment and should be considered as a differential diagnosis in resistant malignancy. One should always take into account potential PET-positivity of non-malignant tissues, e.g., enchondroma that could erroneously lead to upstaging and overtreatment. Finally, additional biopsies and histological verification are warranted in cases of persistent ^18^FDG-avid uptake.
